# Automated analysis of intraoperative phase in laparoscopic cholecystectomy: A comparison of one attending surgeon and their residents

**DOI:** 10.1016/j.jsurg.2023.04.010

**Published:** 2023-07

**Authors:** Gemma Humm, Adam Peckham-Cooper, Ayman Hamade, Christopher Wood, Khaled Dawas, Danail Stoyanov, Laurence B Lovat

**Affiliations:** ⁎ Wellcome/ Engineering and Physical Sciences Research Council Centre for Interventional and Surgical Sciences, University College London, London, United Kingdom; † UCL Division of Surgery and Interventional Science, University College London, London, United Kingdom; ‡ Leeds Institute of Emergency General Surgery, St James University Hospital, Leeds, United Kingdom; § Department of General and Colorectal Surgery. East Kent University Hospitals NHS Foundation Trust, Queen Elizabeth the Queen Mother Hospital, Margate, United Kingdom

**Keywords:** Laparoscopic cholecystectomy, automated video analysis, intraoperative phase, critical view of safety, surgical training, Medical Knowledge, Practice-Based Learning and Improvement, Systems-Based Practice

## Abstract

**Objective:**

This study compares the intraoperative phase times in laparoscopic cholecystectomy performed by an attending surgeon and supervised residents over 10-years to assess operative times as a marker of performance and any impact of case severity on times.

**Design:**

Laparoscopic cholecystectomy videos were uploaded to Touch Surgery™ Enterprise, a combined software and hardware solution for securely recording, storing, and analysing surgical videos, which provide analytics of intraoperative phase times. Case severity and visualisation of the critical view of safety (CVS) were manually assessed using modified 10-point intraoperative gallbladder scoring system (mG10) and CVS scores, respectively. Attending and residents’ times were compared unmatched and matched by mG10.

**Setting:**

Secondary analysis of anonymized laparoscopic cholecystectomy video, recorded as standard of care.

**Participants:**

Adult patients who underwent elective laparoscopic cholecystectomy a single UK hospital. Cases were performed by one attending and their residents.

**Results:**

159 (attending=96, resident=63) laparoscopic cholecystectomy videos and intraoperative phase times were reviewed on Touch Surgery™ Enterprise and analyzed. Attending cases were more challenging (p=0.037). Residents achieved higher CVS scores (p=0.034) and showed longer dissection of hepatocystic triangle (HCT) times (p=0.012) in more challenging cases. Residents’ total operative time (p=0.001) and dissection of HCT (p=0.002) times exceeded the attending's in low-severity matched cases (mG10=1). Residents’ total operative times (p<0.001), port insertion/gallbladder exposure (p=0.032), and dissection of HCT (p<0.001) exceeded the attending's in matched cases (mG10=2). Residents’ total operative (p<0.001), dissection of HCT (p<0.001), and gallbladder dissection (p=0.010) times exceeded the attendings in unmatched cases.

**Conclusions:**

Residents’ total operative and dissection of HCT times significantly exceeded the attending's unmatched cases and low-severity matched cases which could suggest training need, however, also reflects an expected assessment of competence, and validates time as a marker of performance.

## Highlights

• Comparing attending and residents’ intraoperative phase times in laparoscopic cholecystectomy.
• Touch Surgery™ Enterprise provided analytics of intraoperative phase times.
• Residents’ total times can significantly exceed the attending's.
• Residents’ dissection of hepatocystic triangle times can significantly exceed the attending's.
• This could suggest training need and validates time as a marker of performance.

## INTRODUCTION

Total operative time has been used as a surrogate marker of performance in laparoscopic cholecystectomy.^1^^,^^2^ Operative time is influenced by many factors, including patient sex, habitus, underlying pathology, and operative findings. In a retrospective study of 315 laparoscopic cholecystectomies performed by surgical residents in the United States (US), total operative time was significantly longer for junior residents (Post Graduate Year [PGY]1-3) compared to senior residents (PGY4-5), irrespective of the grade of assistant.^1^ Conversely, a study of 71 laparoscopic cholecystectomies found no significant difference in total operative time between junior residents (n=28) 59.6±25.3 minutes, senior residents (n= 28) 56±19.4 minutes and attending surgeons (n=15) 71±37.2 minutes.^2^

Delayed video analysis has been used to assess surgical skills and surgical error as an adjunct to operative time focusing on intraoperative phases within the process of undertaking a laparoscopic cholecystectomy.^3^, ^4^, ^5^, ^6^, ^7^, ^8^, ^9^, ^10^ This process is time consuming and requires teams of expert assessors and as such, until recently, this technique has been used in the research setting only. More recently there have been attempts to digitize a similar process.^11^ It is now possible to digitally analyze surgical video using artificial intelligence to automatically identify intraoperative phases in laparoscopic cholecystectomy.^12^^,^^13^

Using operative time to examine an operation at a more granular level could assist in identifying aspects of cases that require either more focused training attention and/or further training needs. The aim of this study was to compare the total operative time and intraoperative phase timings of surgical residents and an attending surgeon over a 10-year period.

## MATERIAL AND METHODS

### Dataset

The video dataset consisted of fully anonymized operative laparoscopic cholecystectomy videos by a single attending surgeon. Videos were uploaded to Touch Surgery™ Enterprise and anonymized by the RedactOR^TM^ algorithm to ensure any remaining patient identifiable information was removed. RedactOR^TM^ detects portions of the video where the camera is outside of the patient and pixelates the video stream in real-time on upload to prevent the recording of any potentially identifiable information. Operations were undertaken under the care of a single attending surgeon at a UK district general hospital during their first 10-years of practice. Operations were performed by either the attending or their residents. Only full videos of complete cases were included, there were no exclusions of complete cases. Cases had been identified as either attending or resident cases by the attending surgeon at source and saved as such. Resident cases were defined as pooled cases whereby residents were able to log their cases as either “supervised-trainer scrubbed” (STS), “supervised-trainer unscrubbed but in theatre” (STU) and “performed” (P) using the Joint Committee on Surgical Training and Intercollegiate Surgical Curriculum Program trainee supervision codes (See supplementary table 1).^14^^,^^15^ This would therefore allow the attending to log the case as Training a more junior trainee (T). The individual codes STS/STU/P were not recorded by the attending. This video dataset is owned by the attending surgeon, there is no patient/clinical data. Patients provided voluntary, informed consent for their intraoperative video to be used for education and training purposes.

### Touch Surgery™ Enterprise

Touch Surgery™ Enterprise by Digital Surgery™ Ltd, a Medtronic company, is a combined software and hardware solution for securely recording, storing, and analysing surgical videos. Uploaded videos are automatically anonymized by the RedactOR^TM^ algorithm and automatically broken down into phases. Touch Surgery^TM^ phase identification, developed by Digital Surgery Ltd, is based on the state-of-the-art phase recognition models in the literature, which have been previously applied to laparoscopic cholecystectomy,^12^ cataract^14^ and total knee replacement surgery.^15^ The latest model by Digital Surgery™ Ltd achieves a 96% accuracy in detecting phase transitions in laparoscopic cholecystectomy.^16^ Qualified annotators, trained on surgically validated guidelines, quality-assured the model outputs.

Touch Surgery^TM^ defines the surgical workflow phases as the following five operative phases, through liaising with key opinion leaders and consulting the literature:

The following phases are identified:

• P1 - Port insertion/gallbladder exposure
• P2 - Dissection of the Hepatocystic Triangle
• P3 - Ligation and division of cystic artery and cystic duct
• P4 - Gallbladder dissection
• P5 - Specimen removal and removal of ports

### Laparoscopic Cholecystectomy Severity Score

Grading scores often require clinical, biochemical, and radiological data in addition to intraoperative findings and are often used to predict conversion to an open operation. The G10 score is a 10-point intraoperative gallbladder scoring system, which uses an interoperative severity grading of cholecystitis as a marker of technical difficulty (Supplementary table 2), where 0 is the least challenging and 10 the most challenging.^17^ 1 point is scored for a Body Mass Index (BMI) of more than 30. This point was omitted in our study, due to a lack of clinical information. Total scores, out of a maximum of 9, were considered in this study and are referred to as “modified G10” (mG10).

### Visualisation of the Critical View of Safety (CVS)

Appropriate visualization of the CVS prior to ligation and division of the cystic duct and artery are crucial for preventing common bile duct injuries.^18^ An intraoperative score (Supplementary table 3) was devised, which identifies the three domains required to achieve the CVS: Two structures connected to the gallbladder, cystic plate clearance and hepatocystic triangle (HCT) clearance, with scores of 0-2 awarded in each domain. A score of ≥4 represented adequate visualization. However, when reviewers were asked if it was safe to divide, there was no significant agreement across adequate and inadequate scores.^19^ For this study, we used the total score only to indicate the visualization of the CVS.

### Data Extraction: Manual Assessment of Surgical Video

After videos were uploaded to Touch Surgery^TM^ Enterprise, the videos were assessed for completeness and manually scored for intraoperative severity using the mG10 and visualisation of the CVS scores. All videos were assessed by GH, a clinical research/training fellow and general surgery resident who holds Membership of the Royal College of Surgeons and APC a consultant surgeon who holds Fellowship of the Royal College of Surgeons. The first 50% of complete video cases were also assessed by an attending surgeon and interrater reliability (IRR) analysis was performed.

### Data extraction: Automated Identification of Intraoperative Phase

Touch Surgery™ Enterprise provided a .csv file with case and phase durations in hours and minutes (hh:mm). Operative times were converted to minutes (decimal).

### Statistical Analysis

Data was analyzed using descriptive, nonparametric statistics and variables were analyzed for positive relationships using linear regression in GraphPad Prism 9 for MacOS Version 9.3.1 (350), December 7, 2021. IRR was analyzed using ICC two-way mixed effects model in using IBM SPSS Statistics Version 27, 2020. Qualitative interpretations of ICC values recommended “poor” (ICC < 0.5), "moderate" (0.5–0.75), “good” (0.75–0.9) and "excellent" (ICC > 0.9).^20^^,^^21^

## RESULTS

159 complete laparoscopic cholecystectomy videos were analysed (attending=96, resident=63).

### Interrater Reliability

Good agreement was found between the two raters for mG10 score (ICC=0.800, 95% CI=0.511-0.897 and moderate agreement (ICC=0.675, 95% CI=0.077-0.85) achieved for CVS scores as shown in Supplementary table 4.

### Case Selection

Rater assessment using mG10 score was significantly higher in the attending's cases (Attending: median=2, IQR=2 range=0-6), Resident: median=1, IQR=1, range=0-5, Mann Whitney U=2454, p=0.037). No significant difference between attending and resident CVS scores were identified.

### Intraoperative Phase Times and mG10 and CVS Score

**Figure 1:**
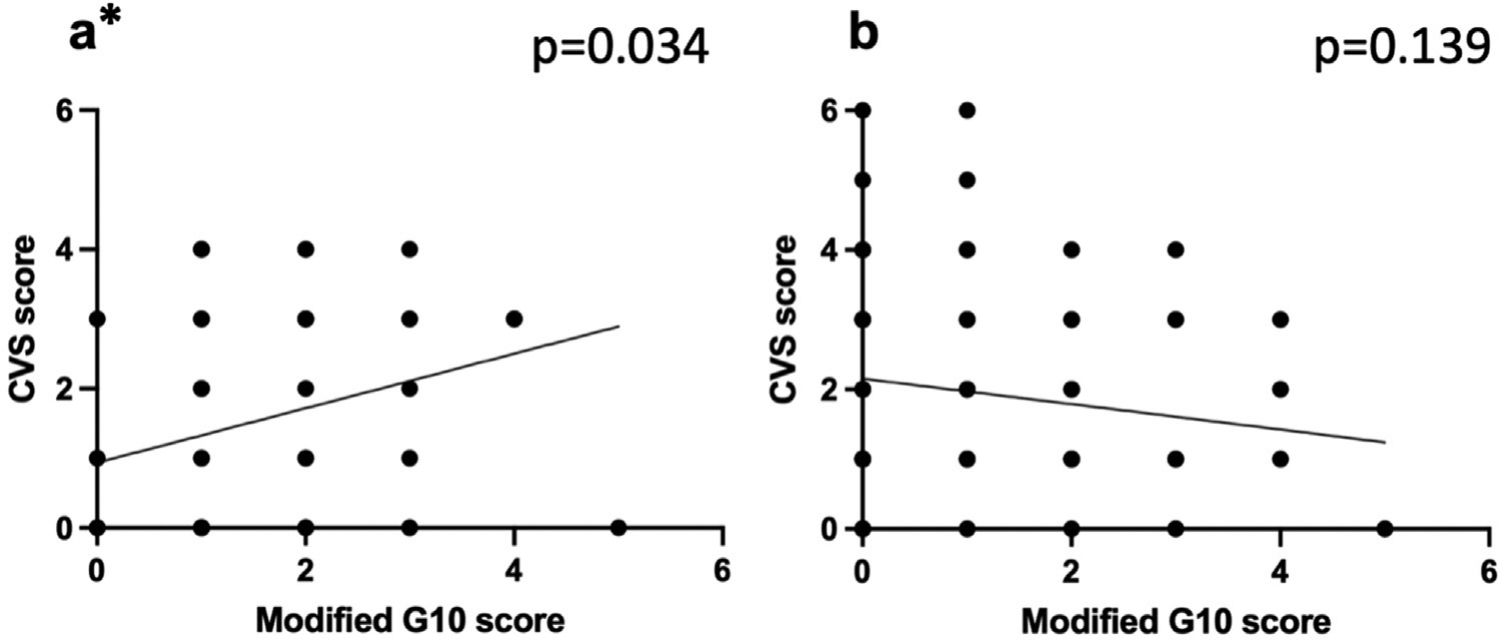
Scatter plots and linear regression analysis of G10 score and Critical View of Safety (CVS) score for (1a)residents and (1b) attending. * marks statistically significant results.

**Figure 2:**
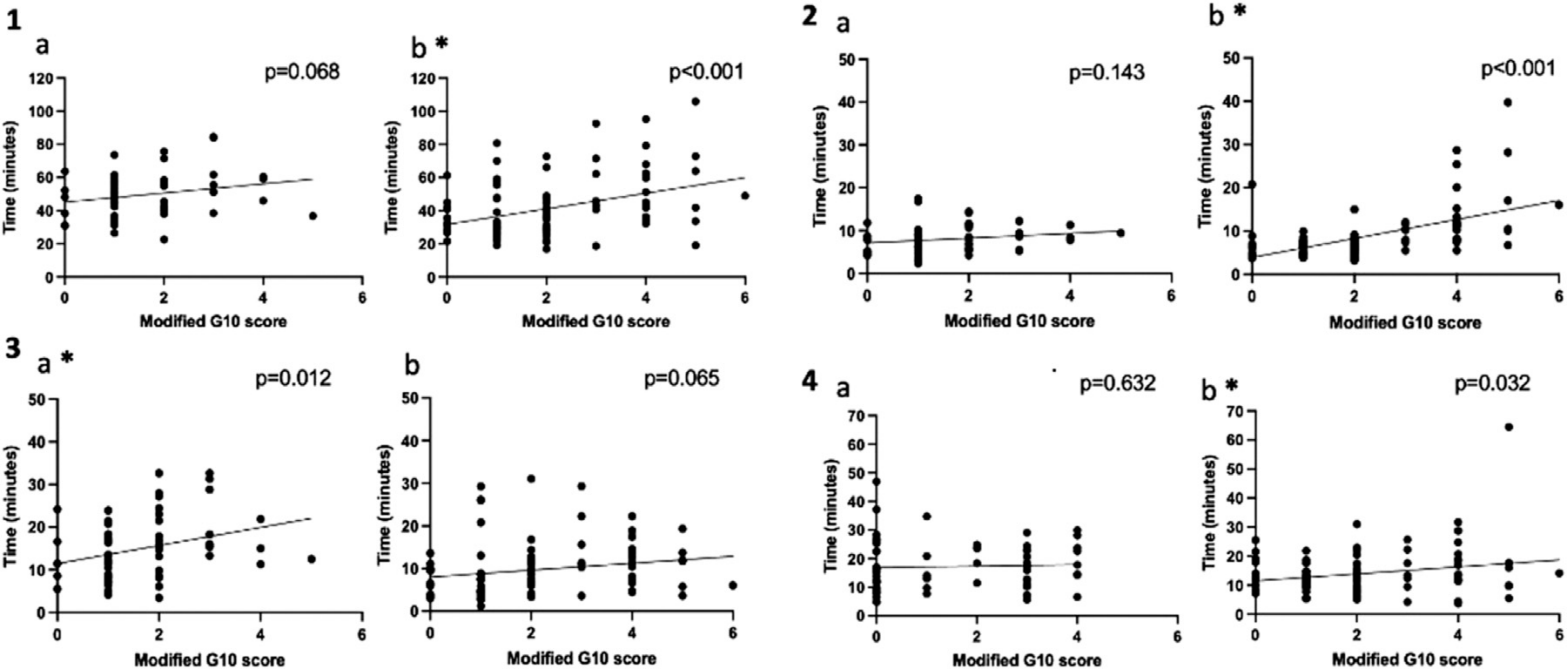
Scatter plots and linear regression analysis of modified G10 (mG10) and operative times for (2.1a) Resident mG10 score (mG10) vs total operative time 1b Attending mG10 score vs total operative time. (2.2a) Resident mG10 vs P1 operative time (2.2b) Attending mG10 vs P1 operative time. (2.3a) Resident mG10 v P2 operative time. (2.3b) Attending mG10 v P2 operative time. (2.4a) Resident mG10 v P4 operative time. (2.4b) Attending mG10 v P4 operative time. * marks statistically significant results.

**Figure 3:**
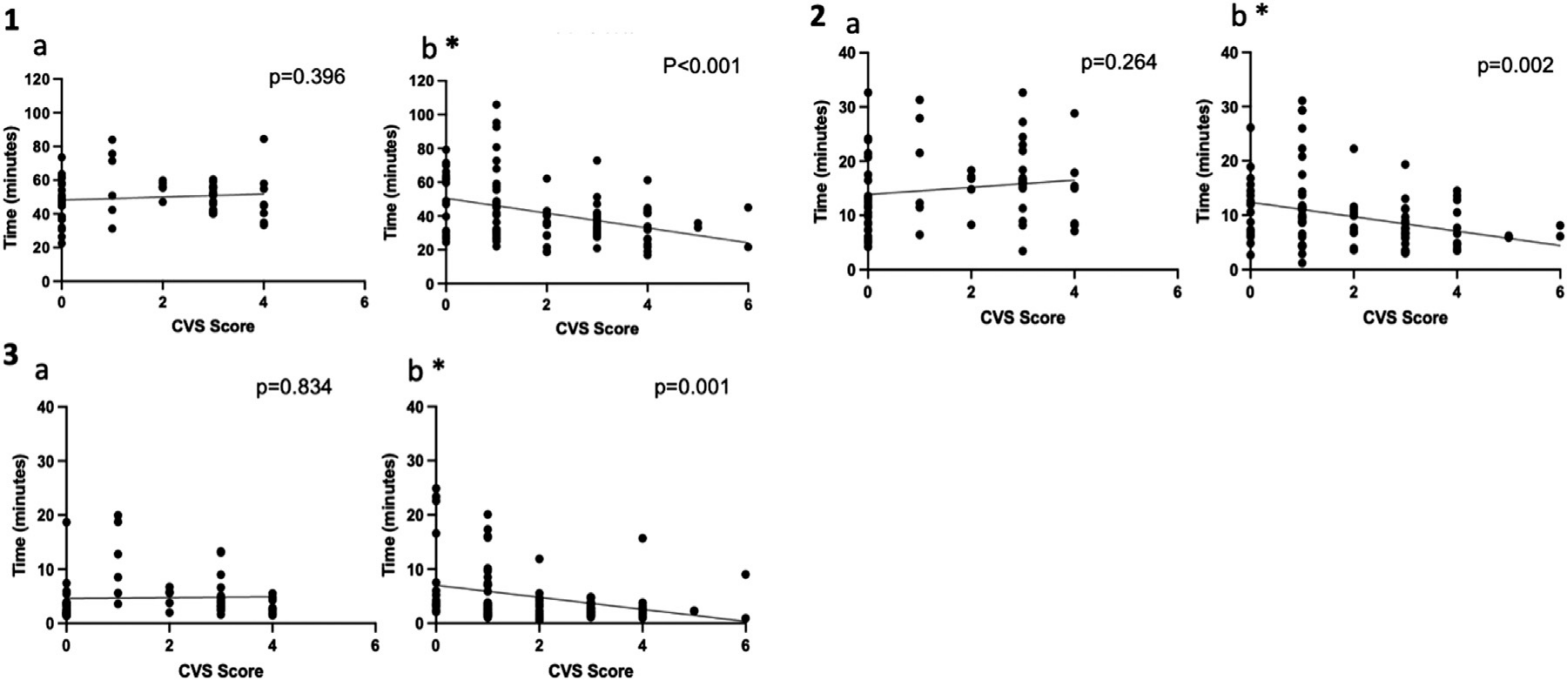
Scatter plots and linear regression analysis of Critical View of Safety Score (CVS) and operative times for (3.1a) Resident CVS score vs Total operative time. (3.1b) Attending CVS score vs Total operative time. (3.)2a Resident CVS score vs P2 operative time. (3.2b) Attending CVS score vs P2 operative time. (3.3a) Resident CVS Score vs P3 operative time. (3.3b) Attending CVS score v P3 operative time. * marks statistically significant results.

A higher mG10 score represents a more challenging cases and a higher CVS score represents a better visual visualization of the CVS. In resident cases a positive relationship between rater assessed mG10 and CVS scores (r^2^=0.071 p=0.034) was found, but not in the attending's cases (r^2^=0.023, p=0.136), as shown in Figure 1. Figure 2 shows the intraoperative phase times which have significant positive linear relationships with the mG10 scores. Positive relationships were found between mG10 score and P2 (r^2^=0.098 p=0.012) in resident cases, but not in the attending's cases. In the attending's cases positive linear relationships were found between mG10 score and P1 (r^2^=0.311 p<0.001), P4 (r^2^=0.047 p=0.032) and total operative times (r^2^=0.147 p<0.001). Figure 3 shows intraoperative phase times which have significant relationships with CVS score. In the attending's cases significant negative relationships were found between CVS score and P2 (r^2^=0.101 p=0.001), P3 (r^2^=0.104 p=0.001) and total operatives times (r^2^=0.134 p<0.001). There were no significant relationships found between CVS score and operatives times in resident cases.

### Unmatched Cases

**Figure 4:**
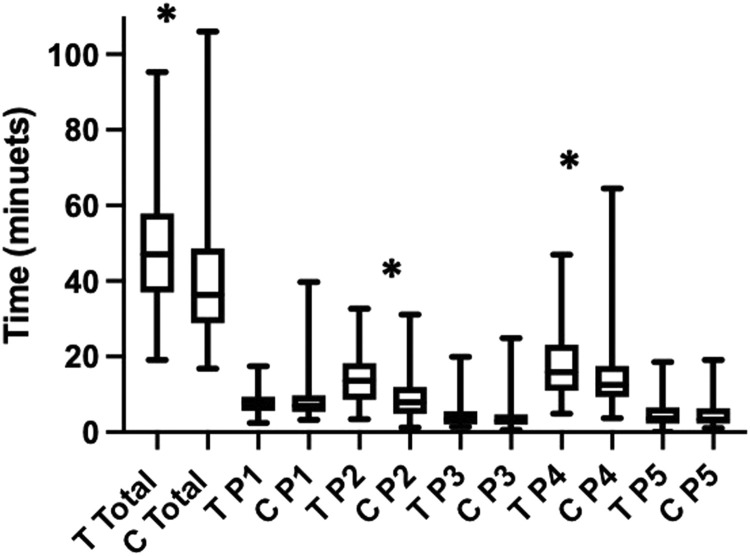
Box and whisker plot showing median, interquartile range and range of total and intraoperative phase operating times for residents and attending for all cases, unmatched. (T Resident, C Attending, P1 Port insertion/gallbladder exposure, P2 Dissection of the Hepatocystic Triangle, P3 Ligation and division of cystic artery and cystic duct, P4 Gallbladder dissection, P5 Specimen removal and removal of ports). * marks statistically significant results.

Table 1 shows the median, IQR, range and Mann-Whitey U statistics of residents’ and the attending's total operative and intraoperative phases times of unmatched cases. Figure 4 shows box and whisker plots for residents’ and the attending's total operative and intraoperative phases times of unmatched cases. Significant differences were found between residents’ and the attending's total operative times (U=3199, p<0.001), P2 (U=1624 p<0.001) and P4 (U=2295, p=0.010). No significant differences were found between the remaining intraoperative phase times.

**Table 1:** A Comparison of Consultant and Trainee Total and Intraoperative Phase Operating Times for Unmatched Cases

	Unmatched Cases Trainee (n=63)	Consultant (n=96) Median, IQR (range)	Mann-Whitney U
Total operative time	47.1, 20.9 (95.3-19.1)	36.4, 19.9 (16.8-106)	**3199 (p<0.001)**
Port insertion/exposure	7.87, 3.83 (2.4-17.4)	7.01, 4.38 (3.22-39.70	2775 p=0.382
Dissection of HCT	13.6, 9.75 (3.45-32.7)	7.92, 7.04 (1.22-31.1)	**1642 p<0.001**
Ligation/division of cystic structures	3.42, 3.49 (1.38-20)	2.96, 24.9 (0.633-24.9)	2722 p=0.288
Gallbladder dissection	15.9, 12.1 (4.87-47)	12.5, 8.33 (3.73-64.5)	**2295 p=0.010**
Specimen out/closure	3.65, 4.35 (0.033-18.6)	3.37, 4.14 (1.08-19.1)	3000 p=0.933

Statistical significance indicated in bold.

### Case Matching

Attending and resident cases were matched by mG10 score. No significant differences in intraoperative times were found when cases were matched by mG10 scores of 0, 3 and 4. There were insufficient cases to match for mG10 scores of 5 and 6.

**Figure 5:**
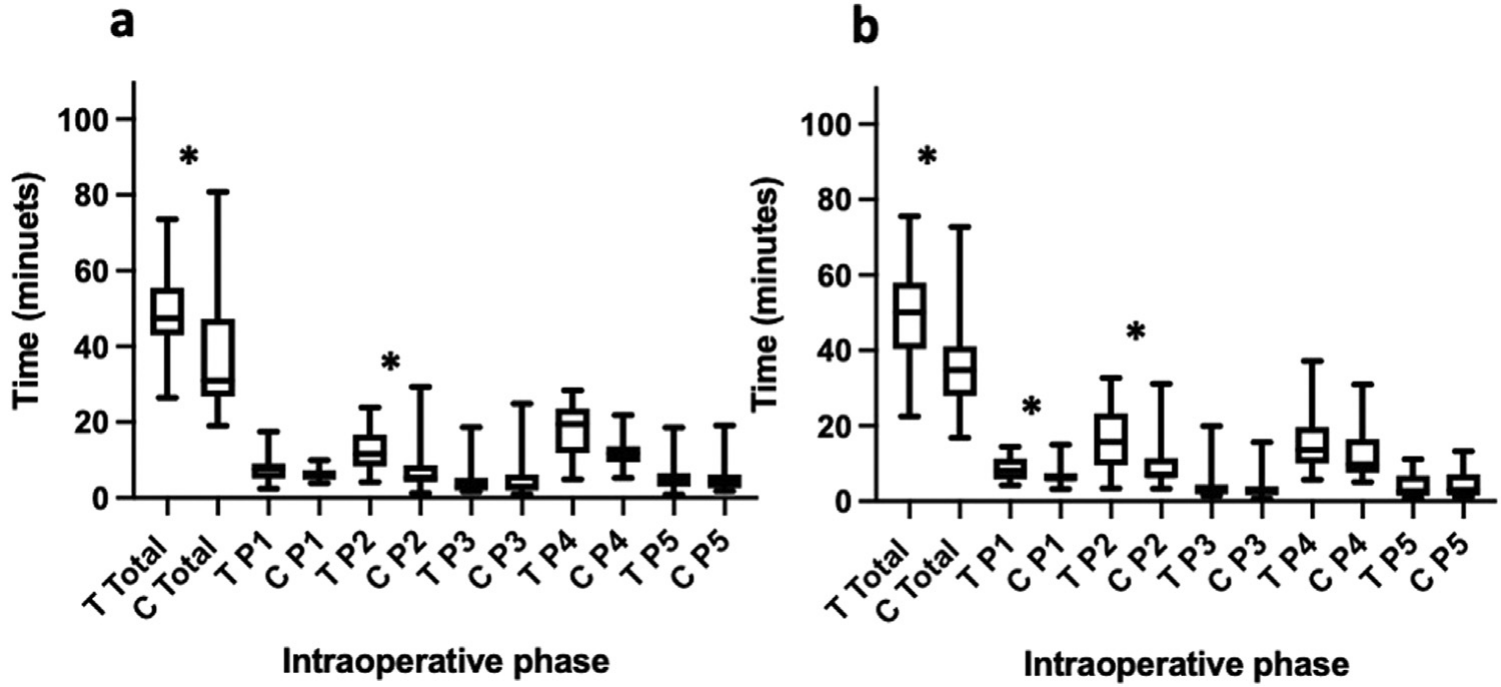
Box and whisker plots showing median, interquartile range and range of total and intraoperative phase operating times for residents and attending for cases matched by (5a) mG10=1 and (5b) modified G10=2. (T Resident, C Attending, P1 Port insertion/gallbladder exposure, P2 Dissection of the Hepatocystic Triangle, P3 Ligation and division of cystic artery and cystic duct, P4 Gallbladder dissection, P5 Specimen removal and removal of ports). * marks statistically significant results.

Table 2 shows the median, IQR, range and Mann-Whitey U statistics of residents’ and the attending's total operative and intraoperative phases times of cases matched by mG10 score. Figure 5 shows the box and whisker plots for residents’ and the attending's total operative and intraoperative phases times with matched cases for mG10 scores 1 and 2.

**Table 2:** A Comparison of Consultant and Trainee Total and Intraoperative Phase Operating Times for Cases Matched by Modified G10 (mG10) Score.

	Matched mG10=0 Trainee (n=6)	Consultant (n=11) Median, IQR (range)	Mann-Whitney U
Total operative time	43.2, 23.9 (30.8-63.7)	32.2, 13.4 (21.5-61.3)	22 (p=0.301)
Port insertion/exposure	6.55, 4.83 (4.23-11.8)	6, 2.55 (3.78-20.8)	27 (p=0.590)
Dissection of HCT	11.5, 10.71 (5.5-24.2)	9.48, 6.05 (3.07-13.6)	27 (p=0.590)
Ligation/division of cystic structures	3.3, 2.55 (1.47-5.95)	2.58, 1.99 (1.38-4.77)	25 (p=0.462)
Gallbladder dissection	12.9, 15 (9.13-47)	14.1, 9.18 (7.2-25.5)	30 (p=0.807)
Specimen out/closure	2.12, 2.68 (0.033-2.69)	2.73, 1.79 (1.4-7.2	19 (p=0.180)
	Matched mG10=1		
	Trainee (n=28)	Consultant (n=26)	Mann-Whitney U
		Median, IQR (range)	
Total operative time	47.4.6, 12.6 (26.5-73.6)	30.9, 20.6 (19-80.8)	**180 (p=0.001)**
Port insertion/exposure	7.62, 4.05 (2.4-17.4)	6.07, 2.55 (3.92-9.92)	262.5 (p=0.076)
Dissection of HCT	11.6, 8.45 (4.2-23.9)	5.38, 4.46 (1.22-29.3)	**189.5 (p=0.002)**
Ligation/division of cystic structures	3.06, 3.26 (1.62-18.7)	2.96, 4.14 (0.983-24.9)	354 (p=0.871)
Gallbladder dissection	19.5, 11.8 (4.87-23.5)	11.8, 4.22 (5.25-21.9)	354 (p=0.871)
Specimen out/closure	4.26, 3.55 (0.833-18.6)	4.32, 3.57 (1.83-19.1)	347.5 (p=0.780)
	Matched mG10=2		
	Trainee (n=18)	Consultant (n=29)	Mann-Whitney U
		Median, IQR (range)	
Total operative time	50.1, 17.6 (22.5-75.6)	34.8, 1.2 (16.8-72.7)	**99 (p<0.001)**
Port insertion/exposure	8.08, 5.57 (4.23-14.5)	6.15, 2.09 (3.22-15)	**163.5 (p=0.032)**
Dissection of HCT	15.7, 13.9 (3.45-32.7)	6.98, 5.23 (3.37-3.1)	**105 (p<0.001)**
Ligation/division of cystic structures	3.18, 2.57 (1.4-20)	3.1, 2.43 (0.717-15.7)	222 (p=0.400)
Gallbladder dissection	13.6, 9.5 (5.7-37.20)	9.78, 8.9 (5.7-31)	201 (p=0.194)
Specimen out/closure	2.71, 5.34 (0.783-11.1)	3.02, 5.73 (1.15-13.3)	236 (p=0.591)
	Matched mG10=3		
	Trainee (n=7)	Consultant (n=7)	Mann-Whitney U
		Median, IQR (range)	
Total operative time	55.4, 33 (38.5-84.5)	45.9, 30.8 (18.7-92.6)	20 p=0.620
Port insertion/exposure	9.13, 3.93 (5.33-12.2)	10.3, 423 (5.58-12.1)	19 p=0.535
Dissection of HCT	18.3, 15.9 (13.3-32.7)	11.4, 11.9 (3.6-29.3)	11 p=0.097
Ligation/division of cystic structures	5.58, 5.55 (2.37-13.1)	5.53, 13.95 (0.633-22.6)	24 >0.999
Gallbladder dissection	15.3, 16.6 (10.3-34.8)	13.4, 12.9 (4.2-25.7)	21 p=0.710
Specimen out/closure	4.82, 4.48 (1.68-12.9)	7.15, 5.92 (1.62-9.52)	24 p=>0.999
	Matched mG10=4		
	Trainee (n=3)	Consultant (n=16)	Mann-Whitney U
		Median, IQR (range)	
Total operative time	59.2, 14.5 (46-60.5)	48.1, 24.8 (32.2-95.3)	21 p=0.792
Port insertion/exposure	8.42, 3.47 (7.83-11.3)	11.5, 6.55 (5.57-28.7)	14 p=0.303
Dissection of HCT	15, 10.6 (11.3-21.9)	11, 7.36 (4.48-22.3)	12 p=0.210
Ligation/division of cystic structures	4.37, 4.13 (2.5-6.63)	3.07, 7.63 (0.783-23.4)	23 p=0.957
Gallbladder dissection	24.4, 19 (10.2-19)	15.3 12.1 (3.73-31.7)	19 p=0.633
Specimen out/closure	4.2, 2.33 (2.97-5.3)	4.38, 4.98 (1.08-13.3)	22 p-0.875

Statistical significance indicated in bold.

### Modified G10 Score of 1

Significant differences were found between residents’ and the attending's total operative times (U=180, p=0.001) and phase time, P2 (U=189 p=0.002). No significant differences were found between the remaining intraoperative phase times.

### Modified G10 Score of 2

Significant differences were found between residents’ and the attending's total operative times (U=99, p<0.001) and phase times, P1 (U=1635 p=0.032) and P2 (U=105, p<0.001). No significant differences were found between the remaining intraoperative phase times.

### Intraoperative events

During manual review of the laparoscopic cholecystectomy videos any intraoperative events which could potentially increase operative times were identified. Cases with total or intraoperative phase times outlying the IQR were reviewed for documented intraoperative events. Five outlying resident cases were identified. One case required considerable adhesiolysis, a further case was prolonged because of a minor cystic duct injury (requiring no further intervention) and finally 3 cases were impacted by iatrogenic intraoperative gallbladder perforations. Similarly, the single outlying attending case resulted from an iatrogenic gallbladder perforation. There were no cases analysed that necessitated conversion to open cholecystectomy.

## CONCLUSIONS

Touch Surgery™ Enterprise anonymizes, stores, and breaks down recorded procedures into intraoperative phases and timestamps for analysis, allowing the investigation of outlying cases and intraoperative phases. The anonymized video dataset used in this study had no accompanying clinical information, therefore the mG10 score was used in this study to manually assess the clinical severity of each case. This score is influenced by the presence of intra-abdominal adhesions and the appearance of the gallbladder,^17^^,^^22^ which can be secondary to previous surgery, previous infection (e.g., cholecystitis, cholangitis) and/ or pancreatitis and can present challenges to abdominal access and laparoscopic placement. Chronic and repeated acute infection/inflammation can fibrose the gallbladder wall obscuring dissection planes, particularly between the gallbladder and liver.^23^ Potentially any of the included cases could have been underscored by 1 point, as BMI was not available. Both intra-abdominal and subcutaneous fat can challenge laparoscopic surgery, and neither could be reliably commented on in this study. This clinical information would normally be available to the surgeon prior to operating and may influence an attending's decision to allow their resident to perform whole or part of an operation. This study found that the median mG10 scores of residents’ cases was lower than attending cases. However, both media scores were of low severity. This study could be underestimating this difference if the attending's case selection included high BMI cases. The data on the previous experience of the resident, training grade and level of attending supervision was not available and could contribute to type II errors. It is likely that residents with less experience will have more attending supervision and have longer operating times compared to more experienced residents.

This study did, however, confirm that over a 10-year period resident total operative time was significantly longer than an attending performed surgery. This was similarly reflected in the important intraoperative phases: dissection of the HCT (P2) and gallbladder dissection (P4). In low-severity (mG10=1,2) matched cases residents perform significantly longer total operative times, laparoscopic port insertion/gallbladder exposure (P1) and dissection of HCT (P2) phase times compared to their attending. This is perhaps not surprising as dissection of the HCT is considered the most crucial phase of laparoscopic cholecystectomy, as isolating the cystic artery and cystic duct, and achieving the CVS are considered crucial to reducing the risk of CBD injury^18^ and performing a technically safe and robust operation. HCT phase has been found to have a higher risk of intraoperative error by attendings.^8^ This study defined and classified surgical errors as consequential and inconsequential.^8^ Consequential errors are any action or omission that resulted in a negative consequence or increased the time of the surgical procedure by necessitating a corrective action. Inconsequential error was described as an action or omission that increased the likelihood of negative consequence and under slightly different circumstances could have had a consequential effect.^8^ This delayed video analysis study of 200 laparoscopic cholecystectomy cases performed by attending surgeons in the UK identified significantly higher frequency of surgical errors in task zone 2 (6.5±5.4), which includes dissection of HCT and ligation and division of cystic structures, compared with task zone 1 (2.9±2.8, p<0.001) and task zone 3 (5.1±3.9 p<0.05).^8^ In the simulated setting surgical residents were found to have an error probability of 7.7% in “dissection of the cystic artery”,^24^ which was lower than “division of the cystic artery and duct’ (15.2% and “separation of the gallbladder from the liver bed” [5.6%]).^24^ This study of 60 ex-vivo porcine laparoscopic cholecystectomies performed by 60 surgical residents in their first year of surgical training also showed significant variation in the number of errors enacted by the subjects.^24^ Whilst, interpreting the findings of these studies in relation to this study should be done with caution, the definitions of intraoperative phases or task zones are not directly comparable, with definitions overlapping across phases (e.g., task zone 2 for attendings in on study^8^ includes both “dissection of the cystic artery” “division of the cystic artery and duct’ in the other study.^24^ But the prolonged P2 intraoperative phase time in our study could be because of a higher rate of surgical errors by surgical residents compared to the attending. It is however interesting therefore that surgical residents achieved significantly better visualization of the CVS in higher mG10 cases although it did take residents longer to achieve the CVS in cases potentially because of these surgeons being more cautious in these more challenging surgical cases. For the attending's cases, longer P2 was also related to significantly clearer visualization of CVS. These findings of longer P2 times could also reflect surgeons’ knowledge and experience that more challenging cases have a higher risk of bile duct injury and therefore additional care and attention has been spent to achieve the CVS. Making assumptions around cause and effect in these cases is difficult as there remains a huge number of intraoperative and interpersonal confounding factors, therefore, it is important not to label the cause of a prolonged total and P2 intraoperative time phase time as just the result of less developed skill or competence for example. The increase in intraoperative times is undoubtedly multifactorial and beyond the scope of this study to details further but is likely the result of relative inexperience and exposure which could be improved by additional training.

It is equally important to consider the matched cases where no significant differences were found between residents’ and attending operative times in cases matched by mG10 scores of 0, 3 and 4. There were insufficient cases with higher mG10 scores for analysis, which likely reflects the national disease severity distribution at the time of data collection and/or individual operator patient selection. A mG10 score of 0 reflects the most straightforward of cases. No significant differences were found between resident and attending operative times. Nevertheless, without understanding resident experience and attending supervision in resident cases it is difficult interpret these results. However, it is broadly acknowledged that laparoscopic cholecystectomy presents a spectrum of challenge from cases requiring basic laparoscopic skills to those cases requiring advanced laparoscopic skills and biliary surgical expertise. There was also no significant difference between resident and attending operating times for moderately challenging cases, (e.g., mG10 3-4), Again conclusions are difficult to extrapolate but this could signify that those cases are challenging for everyone and are somewhat related to whether the resident has autonomy throughout the case. This could also suggest that there is an optimal level of case difficulty for training, with a training potential present in cases with a mG10 score of 2-3.

This study also reported a positive linear relationship between mG10 score and time for port insertion/exposure (P1), gallbladder dissection (P4) and total operative time for the Attending's cases. This finding was not seen in the resident group. However, there was a positive linear relationship between mG10 score and dissection of HCT (P2) in the resident case cohort. It is possible that this relationship was not seen in the resident cases due to attending case selection. Conversely, these findings could also suggest that an increase in case severity presents different challenges to residents and attendings or increase anxiety in residents promoting over caution. It is not possible to identify the reasons for the difference in intraoperative phase times from the findings of this study. The findings may support the view that the dissection of HCT is a challenging phase to master and focusing cognitive, technical, and perhaps simulation skills training in this intraoperative phase could improve resident's dissection of HCT phase time, which in turn could improve total operative times.

In this study, residents tended to achieve greater visibility of the CVS in more challenging cases, whilst similar CVS scores were achieved throughout the spectrum of attending's cases. It could be that residents focused more on achieving adequate CVS when cases were more challenging because of the increased associated risk. This may reflect resident's fear and apprehension around this area of dissection. Despite this, there was no significant differences identified between median resident CVS score and those of the attending cases.

The authors acknowledge that this study has limitations. The anonymized video dataset contains no clinical data, nor data on the grade/experience of the operating surgical resident, assisting surgeon or supervision code (STS/STU/P) for the case. Whilst case severity was inferred using the mG10 score, it was not possible to analyse other perioperative risk factors other perioperative factors that influence case severity or postoperative complications. Pooled analysis of all residents was undertaken on the assumption that residents have a similar level of competence and experience and therefore are likely to have the similar training needs and baseline competence. Whilst this may have introduced some type II error, particularly if an intraoperative phase was largely performed by the attending, it does provide a useful comparable dataset from which conclusions can be drawn.

It would not be appropriate to assume the extremes that the resident has full autonomy in all cases, nor performed a negligible proportion of all cases. It is likely that there is a mix which reflects the diversity of supervision that exists in a population of residents and is appropriate for training cases. The JCST and ISCP trainee supervision codes gives clear guidance on the definitions of supervision and the logging of cases; based on the P, STS, STU criteria it is presumed that significant components of each case were performed by the resident. Additionally, If a resident were assisting in a case this would be logged as such, in keeping with the supervision code Assisting (A): trainee scrubbed to assist, but not taking a leading part in the operation itself, e.g. in the deception, anastomosis.).^14^^,^^15^ Unfortunately, the detailed supervision code was not recorded at source. However, this dataset includes all the attending's in their 10-year practice, reducing the risk of a reporting bias and offering a representation of resident case selection at the time. As this video dataset is fully anonymized it is not possible to retrospectively identify the resident involved in the case. This data would have enriched the dataset and allowed more detailed conclusion but is limitation of secondary research.

Nonetheless, this study confirms residents had longer total operating time with a particular focus of additional time in the P2 phase. As discussed, broad conclusions are difficult, but we would suggest that identifying this area as a focus for more selective could result in an improved P2 performance time and reduce total operating time as a result. Utilization of this tool therefore works to identify specific intraoperative areas of training focus with which to target either individual residents if a video logbook was maintained or a broader selective course curriculum. The future utility for improved training programmes and identification of resident surgical strengths and weaknesses holds potential.

This video dataset is the work of a single attending and their residents and can only infer interpretations on those included. At present this cannot be generalized to the wider population of residents and trainers. Future research should include prospectively collected video datasets with comprehensive operative and clinical data from multiple units to allow comparisons and gain richer insights. Additional data should be collected on resident experience and supervision codes to allow more granular assessment, which should support the development of deeper conclusions. There remains a significant governance argument for recording and storing all laparoscopic cases as visual operation notes. This would provide a huge potential future database and research resource for further work.

## Disclosures

GH received funding from Medtronic (Digital Surgery Ltd is a subsidiary of Medtronic) to undertake this work.

DS is an employee of Digital Surgery Ltd.

The remaining authors have no conflict of interest to declare.

This manuscript is being submitted for consideration of publication in Journal of Surgical Education as an original article. This manuscript has not been submitted for publication elsewhere. Data from this study was presented as SAGES 2021 as a poster presentation.

## Data statement

The datasets generated during and/or analyzed during the current study are available from the corresponding author on reasonable request.

## Acknowledgements

The authors would like to thank and acknowledge Digital Surgery Ltd, a Medtronic company, for access to Touch Surgery TM Enterprise for both technical support, video storage, anonymisation, and phase analysis throughout this study, for the use of their platform and technical support and Dr Christina Fleming and Dr Helen Mohan for proofreading.

## Appendix SUPPLEMENTARY INFORMATION

Image, application 1

## References

[1] Kauvar DS, Braswell A, Brown BD, Harnisch M. (2006). Influence of resident and attending surgeon seniority on operative performance in laparoscopic cholecystectomy. J Surg Res.

[2] Wang WN, Melkonian MG, Marshall R, Haluck RS. (2001). Postgraduate year does not influence operating time in laparoscopic cholecystectomy. J Surg Res.

[3] Ahlberg G, Enochsson L, Gallagher AG (2007). Proficiency-based virtual reality training significantly reduces the error rate for residents during their first 10 laparoscopic cholecystectomies. Am J Surg.

[4] Grantcharov TP, Kristiansen VB, Bendix J, Bardram L, Rosenberg J, Funch-Jensen P. (2004). Randomized clinical trial of virtual reality simulation for laparoscopic skills training. Brit J Surg.

[5] Hamilton EC, Scott DJ, Fleming JB (2002). Comparison of video trainer and virtual reality training systems on acquisition of laparoscopic skills. Surg Endosc.

[6] Palter VN, Orzech N, Reznick RK, Grantcharov TP. (2013). Validation of a structured training and assessment curriculum for technical skill acquisition in minimally invasive surgery: a randomized controlled trial. Ann Surg.

[7] Joice P, Hanna GB, Cuschieri A. (1998). Errors enacted during endoscopic surgery—A human reliability analysis. Appl Ergon.

[8] Tang B, Hanna GB, Joice P, Cuschieri A (2004). Identification and categorization of technical errors by Observational Clinical Human Reliability Assessment (OCHRA) during laparoscopic cholecystectomy. Arch Surg.

[9] Eubanks TR, Clements RH, Pohl D (1999). An objective scoring system for laparoscopic cholecystectomy. J Am Coll Surg.

[10] Seymour NE, Gallagher AG, Roman SA, O'Brien MK, Andersen DK, Satava RM (2004). Analysis of errors in laparoscopic surgical procedures: a new methodology. Surg Endosc.

[11] C-SATS. C-SATS. Published 2020. https://www.csats.com/

[12] Twinanda AP, Shehata S, Mutter D, Marescaux J, de Mathelin M, Padoy N. (2017). EndoNet: a deep architecture for recognition tasks on laparoscopic videos. IEEE Trans Med Imaging.

[13] Garrow CR, Kowalewski KF, Li L (2021). Machine learning for surgical phase recognition. Ann Surg.

[14] Zisimopoulos O, Flouty E, Luengo I (2018). DeepPhase: surgical phase recognition in CATARACTS videos. Lecture Notes in Computer Science (including subseries Lecture Notes in Artificial Intelligence and Lecture Notes in Bioinformatics).

[15] Kadkhodamohammadi A, Sivanesan Uthraraj N, Giataganas P (2021). Towards video-based surgical workflow understanding in open orthopaedic surgery. Comput Methods Biomech Biomed Eng Imaging Vis.

[16] Kadkhodamohammadi A, Luengo I, Stoyanov D. (2022). PATG: position-aware temporal graph networks for surgical phase recognition on laparoscopic videos. Int J Comput Assist Radiol Surg.

[17] Sugrue M, Coccolini F, Bucholc M (2019). Intra-operative gallbladder scoring predicts conversion of laparoscopic to open cholecystectomy: A WSES prospective collaborative study. World Journal of Emergency Surgery.

[18] Pucher PH, Brunt LM, Fanelli RD, Asbun HJ, Aggarwal R. (2015). SAGES expert Delphi consensus: critical factors for safe surgical practice in laparoscopic cholecystectomy. Surg Endosc.

[19] Carr BD, Matusko N, Sandhu G, Varban OA. (2018). Cut or do not cut? Assessing perceptions of safety during laparoscopic cholecystectomy using surgical videos. J Surg Educ.

[20] Liljequist D, Elfving B, Roaldsen KS. (2019). Intraclass Correlation – A Discussion and Demonstration of Basic Features. PLoS One.

[21] Koo TK, Li MY. (2016). A guideline of selecting and reporting intraclass correlation coefficients for reliability research. J Chiropr Med.

[22] Sugrue M, Sahebally SM, Ansaloni L, Zielinski MD. (2015). Grading operative findings at laparoscopic cholecystectomy- A new scoring system. World J Emerg Surg.

[23] Beckingham IJ. Gallstones. In: *Hepatobiliary and Pancreatic Surgery: Companion to Specialist Surgical Practice*. Vol 39.; 2019:171-183. 10.1016/j.gtc.2010.02.010

[24] Tang B, Hanna GB, Cuschieri A. (2005). Analysis of errors enacted by surgical trainees during skills training courses. Surgery.

