# Supplementary material for: Automated analysis of intraoperative phase in laparoscopic cholecystectomy: A comparison of one attending surgeon and their residents
Source: J Surg Educ. 2023 Jul;80(7):994–1004. doi: 10.1016/j.jsurg.2023.04.010 (PMC10664073; doi:10.1016/j.jsurg.2023.04.010)
Supplement: Supplementary file 1 [file mmc1.docx]

| Description | Score |
| --- | --- |
| Appearance |  |
| Adhesions <50% of gallbladder | 1 |
| Adhesions > 50% of gallbladder but gallbladder buried | 2 |
| Completely buried gallbladder | 3 |
|  | Maximum score 3 |
| Distension/contraction |  |
| Distended gallbladder or contracted shrilled gallbladder | 1 |
| Unable to grasp with atraumatic lap forceps | 1 |
| Stone >1cm impacted in Hartmann’s pouch | 1 |
| Access |  |
| Body Mass Index >30 | 1 |
| Adhesions from surgery limiting access | 1 |
| Severe sepsis and complications |  |
| Free bile/pus outside of gallbladder | 1 |
| Fistula | 1 |
| TOTAL (MAXIMUM 10) |  |

Supplementary table 2. Intraoperative grade score adapted from Sugrue et al., (2019) (1). Body mass index was not available for this study.

1. Sugrue M, Coccolini F, Bucholc M, Johnston A, Manatakis D, Ioannidis O, et al. Intra-operative gallbladder scoring predicts conversion of laparoscopic to open cholecystectomy: A WSES prospective collaborative study. World Journal of Emergency Surgery. 2019;14(1):10–7.
